# A Follow-Up Study of Ovarian Cancer (OOPS): A Study Protocol

**DOI:** 10.3389/fnut.2022.872773

**Published:** 2022-06-16

**Authors:** Ting-Ting Gong, Fang-Hua Liu, Ya-Shu Liu, Shi Yan, He-Li Xu, Xin-Hui He, Yi-Fan Wei, Xue Qin, Song Gao, Yu-Hong Zhao, Qi-Jun Wu

**Affiliations:** ^1^Department of Obstetrics and Gynecology, Shengjing Hospital of China Medical University, Shenyang, China; ^2^Department of Clinical Epidemiology, Shengjing Hospital of China Medical University, Shenyang, China; ^3^Clinical Research Center, Shengjing Hospital of China Medical University, Shenyang, China; ^4^Key Laboratory of Precision Medical Research on Major Chronic Disease, Shengjing Hospital of China Medical University, Shenyang, China

**Keywords:** ovarian cancer, follow-up, cohort study, survival, diet

## Abstract

The ovarian cancer (OC) follow-up study (OOPS) is an on-going hospital-based large prospective longitudinal cohort study aimed to explore the relationship between pre/post-diagnostic biological, clinical, environmental, and lifestyle factors with focus on the diet and OC prognosis (including drug resistance, relapse, and mortality). Patients recruited during the baseline survey were between 18 and 79 years old, with histologically confirmed OC diagnosis. Their follow-up and medical treatment were conducted at the gynecological oncology ward at Shengjing Hospital of China Medical University, Shenyang, China after 2015. A total of 703 OC patients made up the final OOPS study population. The follow-up stage was conducted in both passive and active modes. In the passive mode, the follow-up was performed by linkage to the Liaoning Providence Center for Disease Control and Prevention every 6 months to obtain health outcome results. The status of lifestyle factors was re-estimated using the same measurements as those in the baseline survey. OC participants in the OOPS study completed a questionnaire and anthropometric examinations. In addition, biological specimens were collected during the baseline survey, which included blood, urine, and stool samples that were stored for further use. This article is intended to serve as an introduction to this project and to provide details for investigators who may be carry out related analysis.

## Introduction

Ovarian cancer (OC) is the third most common gynecologic malignancy with a high incidence and about 239,000 new cases reported annually worldwide (1, 2). Its mortality rate has always ranked first in gynecological malignancies and the 5-year survival rate is still <45% in most countries (3, 4). Not surprisingly, the current state of OC in China is not optimistic, with 55,342 new cases and 37,519 deaths in 2020, which have been increasing each year (5, 6). Three main types of OC have been identified, including epithelial, germ cell, and sex cord-stromal, with epithelial tumors comprising ~95% of all OC cases (7).

Despite remarkable advances in surgery and chemotherapy, prognosis still requires considerable improvement (8). Thus, identifying prognostic factors for OC is critical for reducing its high mortality rates. Over the past decade, a growing number of studies have focused on prognostic factors associated with OC. Some studies have reported that clinical characteristics (e.g., stage at detection, success in optimal debulking, histologic subtype, and chemotherapy) may play critical roles in prognosis (9). Additionally, several demographical and modifiable lifestyle factors (e.g., menopausal hormone therapy use, breastfeeding, psychosocial stress, and diet) can also influence survival in OC (10–17). However, high-quality prognostic studies focusing on these issues have been limited. Furthermore, inconsistent findings have been reported over the course of the recent decade. To gain deeper insight into prognostic factors and to further address inconsistencies related to OC patient survival, a prospective cohort study was conducted at Shengjing Hospital of China Medical University in Shenyang, China.

**Figure 1:**
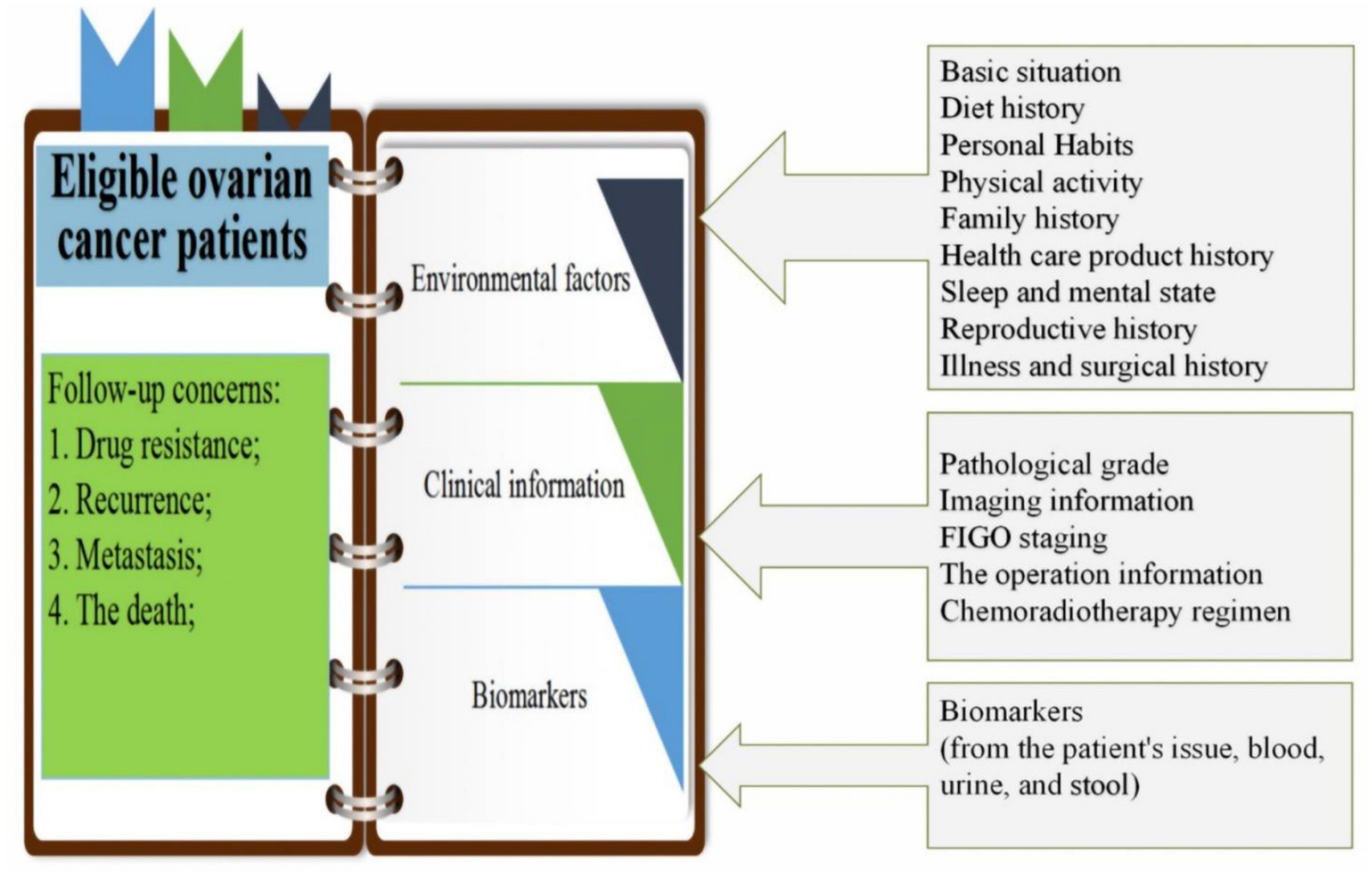
Cohort design for prognosis study of patients with ovarian cancer.

This prospective cohort study included eligible OC patients who provided informed consent for long-term prospective follow-up. The aim of the present study was to investigate the prognostic factors of OC, including patients' clinical information [such as pathological grade, imaging, and International Federation of Gynecology and Obstetrics (FIGO) stage], biomarkers (from the patient's tissue, blood, urine, and stool), and pre/post-diagnostic environmental exposure information (such as personal habits, sleep and mental state, fertility history, diet, physical activity, history of disease and surgery, and family history of chronic diseases) (Figure 1). The cohort was designed to support comprehensive research of the biological, clinical, environmental, and lifestyle factors determining prognosis in OC patients, as well as their inter-relationships, making the present study superior to prior research that only explored the impact of a single factor on the prognosis of OC. The present report describes the study design, provides a cohort description, and outlines preliminary results for factors affecting OC prognosis.

## Methods and Analysis

### Design Overview

**Figure 2:**
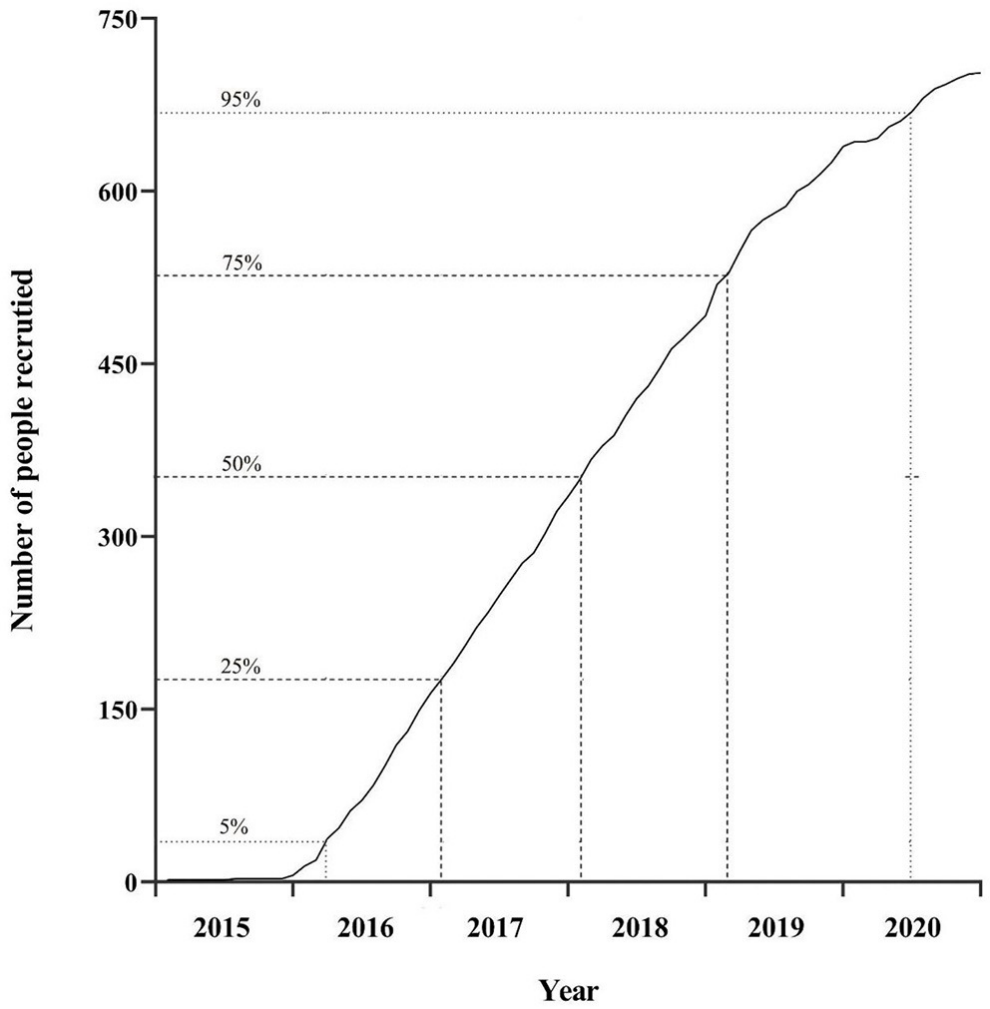
Cumulative recruitment into the cohort.

The ovarian cancer follow-up study (OOPS) is an on-going hospital-based large prospective longitudinal cohort study aimed to explore the relationship between pre/post-diagnostic biological, clinical, environmental, and lifestyle factors with focus on the diet and OC prognosis (including drug resistance, relapse, and mortality). Patients recruited during the baseline survey were between 18 and 79 years old, with histologically confirmed OC diagnosis. Their follow-up and medical treatment were conducted at the gynecological oncology ward at Shengjing Hospital of China Medical University, Shenyang, China after 2015. All patients were able to answer the epidemiological questionnaire. By December 31, 2020, 744 (93%) out of 853 distributed questionnaires were filled out and returned. Of these, 17 OC participants reported a significantly abnormal caloric intake (<500 or >3,500 calories/day) and 24 participants left 11 (10%) or more food items blank, which were excluded. A total of 703 OC patients made up the final OOPS study population. Median recruitment was achieved by the beginning of 2018, with 90% of participants recruited between the beginning of 2016 and the beginning of 2019 (Figure 2).

### Follow-Up Schedules

The follow-up stage was conducted in both passive and active modes. In the passive mode, the follow-up was performed by linkage to the Liaoning Providence Center for Disease Control and Prevention every 6 months to obtain health outcome results. In addition, clinical specialists extracted patient medical data from the information system at Shengjing Hospital every 6 months after the patient finished the baseline survey. This time lapse allowed for definitive staging, pathology evaluation, diagnosis determination, and initial treatment to be completed. In the active mode, all included and surviving OC patients were invited for a face-to-face interview every 6 months. The status of lifestyle factors was re-estimated using the same measurements as those in the baseline survey.

### Data Collection

Ovarian cancer participants in the OOPS study completed a questionnaire and anthropometric examinations. In addition, biological specimens were collected during the baseline survey, which included blood, urine, and stool samples that were stored for further use. Developed written protocols for questionnaires, anthropometric measurements, and biospecimen collection, delivery, and storage were strictly enforced during the baseline survey.

#### Questionnaires

Well-trained interviewers conducted face-to-face interviews using a structured questionnaire to gather information on demographics and socio-economic characteristics, health care product use, health status, reproductive history, diet, individual living habits, passive smoking and indoor air pollution status, physical activity, past medical history, and family history of chronic diseases (Table 1). An average of 45–60 minutes was required to complete a questionnaire. Unqualified questionnaires were excluded from the final analysis. For example, more food items were blank (≥11 items), the same frequency was chosen in most food items.

**Table 1:** Summary of investigations at the baseline survey.

**Investigations**	**No. of variables**	**Variables**
**Questionnaire**		
Demographics and socio-economic characteristics	13	Name, sex, race, national ID number, present address, contact information, occupation, company name, household income, family number, education, marital status, and type of medical insurance
Health care products	12	Vitamin A, vitamin B, vitamin C, vitamin D, vitamin E, multivitamins, calcium, iron, zinc, fish oil/DHA, ginseng, and other dietary supplements
Health status	49	Sleep quality, stress life events, Patient Health Questionnaire (PHQ-9), Generalized Anxiety Disorder scale (GAD-7)
Reproductive history	10	Age of menarche, menopause status, age of menopause, history of menopausal drug use, experience of pregnancy, history of breast feeding, age at parturition of each live birth, duration of breast feeding, experience of assisted reproductive technology, history of intrauterine contraceptive device, history of contraceptive pills use
Diet	131	A 110-item food frequency questionnaire (FFQ), cooking methods of meat, vegetables, and seafoods, dietary habits
Individual living habits	36	Smoking status, age at starting smoking, number of cigarettes per day, depth of smoking, current smoking status, smoking situation today, changes in smoking, drinking status, age at starting drinking, symptoms of drinking, current drinking status, changes in drinking, status of tea drinking, age at starting tea drinking, frequency of changing tea per day, changes in tea drinking, status of carbonated drinks drinking, age at starting carbonated drinks drinking, current status of carbonated drinks drinking, changes in carbonated drinks drinking, status of coffee drinking, age at starting coffee drinking, current status of coffee drinking, changes in coffee drinking
Passive smoking and indoor air pollution	28	Passive smoking exposure, indoor air pollution, pesticide exposure
Physical activity	25	Occupational physical activity, transportation physical activity, leisure-time physical activity, high intensive physical activity, housework, weight changes during last 12 months, weight and height at 25 years old
Past medical history	8	History of disease and history of surgery
Family history	2	Cancers diagnoses and age/dates for first-degree relatives
**Anthropometric measurement**	4	Height, weight, waist circumference, hip circumference
**Clinical information**	5	Histological type, histopathologic grade, FIGO stage, residual lesions, comorbidities

#### Anthropometric Measurements

Well-trained personnel used standard methods to measure patients' height, weight, waist circumference, hip circumference, and blood pressure. Body weight was measured using a digital scale to the nearest 0.1 kg while wearing light clothing and without shoes, after emptying of the bladder. Height was measured without shoes, with stadiometer to the nearest 0.1 cm. Body mass index was calculated using the height and weight measurements. Waist circumference was measured to the nearest 0.1 cm, using plastic tape measure at midpoint between the costal margin and iliac crest in the mid-axillary line, with the subject standing and at the end of a gentle expiration. Hip circumference was measured to the nearest 0.1 cm, at the level of greater trochanters, with the legs close together. Blood pressure after a 5-min rest was measured by a well-trained staff. Two consecutive blood pressure measurements were taken and the arithmetic mean of both measurements were calculated (Table 1).

A clinical nurse was in charge of quality control. He or she checked whether the measurement instruments were in good condition, inspected whether the measurement was in accordance with standard operating procedures, and randomly assigned participants for re-testing.

#### Biorepository

Participants in the OOPS study provided blood, urine, and fecal samples on-site at the time of the baseline survey. Venous blood was collected after overnight fasting (at least 8 h) and mid-stream urine samples were collected. Fecal samples were collected from the middle portion of the feces using a sterile feces collector. The blood samples were aliquoted into 1-ml straws (two straws of each blood and plasma). The urine samples were aliquoted into two 4.5-ml straws. All samples were stored at −80°C in cryogenic refrigerators. Computer systems were used for sample entry, access, and temperature monitoring.

### Ethics and Dissemination

All cohort participants provided written informed consent before the baseline survey. The study was approved by the Institutional Review Board of the Ethics Committee of Shengjing Hospital of China Medical University (2015PS38K). The data would be accessed just by the researchers, who associated with the study and the Ethics Committee. The results of this study will be shown at national and international conferences and peer-reviewed scientific journals. All the results shown in our study will be of group data; thus, individual participants will not be identifiable.

### Patient and Public Involvement

No patients have been involved in the development of the plan for designing, conducting, reporting or implementing this study.

### Statistics

The results are presented as means with standard deviation (SD) for continuous variables and as frequency with percentage for categorical variables. Cox proportional hazards regression was used to calculate the hazard ratio (HR) and 95% confidence interval (CI) for the association of baseline clinical information with overall survival. The proportional hazards assumption was evaluated by including an interaction term between each activity variable and log survival time. No violations were observed (all *P* > 0.05). All analyses were performed using SAS version 9.4 (SAS Institute, Cary, NC, United States). Two-sided *P*-Values of <0.05 were considered statistically significant.

## Results

### Demographics

Table 2 shows the baseline characteristics of OC patients. Participants in this cohort were older, with an average age of 53.63 ± 9.45 years. The Pittsburg Sleep Quality Index was 6.31 ± 3.76. OC patients were assessed using the Patient Health Questionnaire-9 and showed mild depression (6.26 ± 4.72 score). Anxiety in OC patients was assessed using the Generalized Anxiety Disorder scale, which presented normal at 4.16 ± 3.67. More than half of participants had a lower level of education. Similar results were observed for income, with 59.88% (421) with a lower income per month. Almost all participants were insured. Only 7.11% (50) had a family history of cancer. Half of the participants had normal weight. However, above three quarters of them (76.53%) were central obese. And we will (68) have a history of smoking, 21.49% (21.19) have a history of drinking, 32.15% (226) have a history of tea drinking, and over 20% have changed dietary habits or taken dietary supplements. A total of 72.26% (508) went through menopause, and 71.83% (505) had one child or less.

**Table 2:** Demographic information of OOPS from 2015 to June 2020.

**Variables**	**Consented** **(*n* =703)**	**%**
**Age at diagnosis (years)**		
<40	43	6.12
40–49	159	22.62
50–59	288	40.96
60–69	169	24.04
≥69	44	6.26
**Age at diagnosis (years; Mean ±SD)**	53.63 ± 9.45	
**Pittsburg sleep quality index (Mean ±SD)**	6.31 ± 3.76	
**Depression scale scores (Mean ±SD)**	6.26 ± 4.72	
**Anxiety scale scores (Mean ±SD)**	4.16 ± 3.67	
**Age at menopause (Mean ±SD)**	49.73 ± 3.33	
**Educational level**		
Junior secondary or below	375	53.34
Senior high school/technical secondary school	147	20.91
Junior college/university or above	181	25.75
**Income per month (Yuan)**		
<5,000	421	59.88
5,000 to <10,000	194	27.60
≥10,000	88	12.52
**Ever insurance**	681	96.87
**Ever family history of cancer**	50	7.11
**Body mass index (kg/m** ^**2**^ **)**		
<18.5	58	8.25
18.5–23.9	391	55.62
24–27.9	190	27.03
≥28	64	9.10
**Waist-hip ratio**		
<0.85	165	23.47
≥0.85	538	76.53
**Physical activity (MET/h/days)**		
<8	207	29.45
8–15.9	193	27.45
16–23.9	163	23.19
≥24	140	19.91
**Ever diet change**	168	23.90
**Ever cigarette smoking**	68	9.67
**Ever alcohol drinking**	149	21.19
**Ever tea drinking**	226	32.15
**Ever dietary supplement**	150	21.34
**Ever menopause**	508	72.26
**Parity**		
0	5	0.71
1	500	71.12
≥2	198	28.17
**Ever oral contraceptive**	70	9.96

*SD, standard deviation*.

### Diet Information

Diet information for the OC patients is presented in Table 3. Total energy intake for OC patients was 1,455.75 ± 552.64 kcal/day. Staple food consumption was 615.46 ± 233.11 g/day. The intake of meat and eggs was similar at about 37 g/day. The intake amounts of fish and seafood and beans and bean products were 28.52 ± 30.31 and 85.27 ± 78.45 g/day, respectively. Vegetables intake was 214.22 ± 121.72 g/day, which reached the level recommended by the Dietary Guidelines for Chinese residents. However, the intake of fruits was relatively low at 194.64 ± 157.81 g/day. The intake of carbohydrate, fat and protein were 226.90 ± 78.37, 35.04 ± 18.27, and 58.45 ± 24.78 g/day. For vitamins, the highest intake is vitamin C (103.10 ± 62.40 mg/day), the lowest intake is vitamin B12 (0.14 ± 0.21 mg/day). The total fatty acid intake was 9.00 ± 5.72 g/day.

**Table 3:** Diet information of OOPS from 2015 to June 2020.

**Variables**	**Mean**	**Standard**
Total energy intake (kcal/day)	1,455.75	552.64
Staple food (g/day)	615.46	233.11
Meat (g/day)	36.38	29.471
Eggs (g/day)	37.76	27.131
Fish and seafood (g/day)	28.52	30.31
Beans and bean products (g/day)	85.27	78.45
Vegetables (g/day)	214.22	121.72
Fruits (g/day)	194.64	157.81
Carbohydrate (g/day)	226.90	78.37
Fat (g/day)	35.04	18.27
Protein (g/day)	58.45	24.78
Fiber (g/day)	17.51	8.61
Vitamin A (μg/day)	459.93	374.65
Thiamine (mg/day)	0.54	0.27
Riboflavin (mg/day)	0.89	0.41
Niacin (mg/day)	13.66	4.46
Vitamin B6 (mg/day)	0.44	0.22
Vitamin B12 (mg/day)	0.14	0.21
Vitamin C (mg/day)	103.10	62.40
Vitamin E (mg/day)	14.11	9.24
Total fatty acid (g/day)	9.00	5.72

### Clinical Information

Diagnostic information included histological type, histopathologic grade, FIGO stage, residual lesions, and comorbidities (Table 4). A total of 68.14% patients in the cohort had serous OC. In terms of histopathologic grade, 85.21% (*n* = 599) of patients had poorly differentiated OC. There were no residual lesions after surgery in 78.66% (*n* = 553) of patients. A total of 130 (18.49%) deaths occurred before March 31, 2021 during a median follow-up of 37.17 months (interquartile: 24.73–50.17 months).

**Table 4:** Clinical information of OOPS from 2015 to June 2020.

**Clinical variables**	**Consented (*n* = 703)**	**%**
**Histological type**		
Serous	479	68.14
Non-serous	224	31.86
**Histopathologic grade**		
Well-differentiated	56	7.97
Moderately differentiated	48	6.82
Poorly differentiated	599	85.21
**FIGO stage**		
I-II	342	48.65
III-IV	338	48.08
Unknown	23	3.27
**Residual lesions**		
No	553	78.66
<1 cm	106	15.08
≥1 cm	44	6.26
**Comorbidities**	310	44.10
**Vital status**		
Alive	573	81.51
Died	130	18.49

### Clinical Information and Associations With All-Cause Mortality

Non-serous histological subtype, later-stage disease, and greater residual disease were statistically significantly associated with worse survival in this cohort (Table 5). However, an association between histopathologic grade and comorbidities and OC survival was not observed.

**Table 5:** Clinical information and associations with all-cause mortality among OOPS participants.

**Clinical variable**	**No. of deaths/** **total (%)**	**Crude HR** **(95% CI)**	**Adjusted HR^a^** **(95% CI)**
**Histological type**			
Serous	92/479 (19.21)	1.00 (ref)	1.00 (ref)
Non-serous	38/224 (16. 96)	0.87 (0.59–1.27)	1.71 (1.11–2.66)
**Histopathologic grade**			
Well-differentiated	5/56 (8.93)	1.00 (ref)	1.00 (ref)
Moderately differentiated	7/48 (14.58)	1.44 (0.46–4.57)	1.12 (0.35–3.57)
Poorly differentiated	118/599 (19.70)	2.32 (0.95–5.67)	1.76 (0.70–4.43)
**FIGO stage**			
I-II	41/342 (11.99)	1.00 (ref)	1.00 (ref)
III-IV	89/338 (26.33)	2.75 (1.89–4.00)	2.54 (1.65–3.91)
**Residual lesions**			
No	82/553 (14.83)	1.00 (ref)	1.00 (ref)
<1 cm	31/106 (29.25)	2.22 (1.47–3.36)	1.73 (1.11–2.68)
≥1 cm	17/44 (38.64)	3.18 (1.89–5.37)	2.41 (1.39–4.16)
**Comorbidities**			
No	74/393 (18.83)	1.00 (ref)	1.00 (ref)
Yes	56/310 (18.06)	0.82 (0.58–1.16)	0.97 (0.68–1.38)

*CI, confidence interval; HR, hazard ratio; Ref, reference*.

a *Mutually adjusted for all other variables listed in the table*.

### Key Publications

The OOPS study has provided results for diet and OC survival (Table 6). For instance, pre-diagnosis healthy pattern was related to better survival, whereas animal foods pattern was associated with worse survival (18); pre-diagnosis dairy product intake was associated with worse survival (19); pre-diagnosis total cruciferous vegetables and isothiocyanates intake was associated with better survival (20); pre-diagnosis consumption of vitamin B was associated with worse survival (21).

**Table 6:** Previous results for ovarian cancer survival in the OOPS study.

**Reference**	**Journal**	**Year of** **publication**	**Exposure**
(18)	Clinical nutrition	2022	Dietary pattern
(19)	Frontiers in nutrition	2021	Dairy product
(20)	Frontiers in nutrition	2021	Cruciferous vegetables and isothiocyanates
(21)	Frontiers in nutrition	2021	Dietary supplements

## Discussion

As the cohort continues to age, further analyses will be performed to explore the importance of lifestyle and biological characteristics in OC prognosis. This will include studying not only the traditional risk factors, such as cancer characteristics and treatment, but also genetics and other omics, lifestyle (pre/post–prognosis physical activity, diet, passive smoking, and sleep), environmental factors (air pollution and living area conditions), and medical history. And we will apply for funding to ensure the above research proceed. Furthermore, collaborations will be established with nationwide and international institutions to create a multicenter network that includes different types and levels of hospitals. This broad range of participants enables us to generalize results for all patients and thus provides an evaluation of a much wider range of personal health behaviors. Moreover, an in-depth study investigating genetic and molecular profiles will also be conducted. Genome-wide association studies will be carried out to identify novel genetic variants associated with disease development and related phenotypes. Metabolomics (a mass spectroscopy-based technology) techniques will also be available to study the associations between disease status and metabolome. Proteomics will be used to investigate proteins and their post-translational modifications and interactions, providing an opportunity to elucidate complex biological processes and conditions. These studies can lead to a discovery of underlying mechanisms of metastasis, recurrence, and fatality among OC patients. The studies of OC mechanisms will broadly advance the goal of personalized medicine, evidence-based survivorship care, and translation into practical applications, ultimately leading to improvements in public health and healthcare. Finally, artificial intelligence as well as machine and deep learning will be used in future studies to develop integrative risk prediction algorithms for clinical outcome and survivorship endpoints.

The present study collected detailed and comprehensive clinical, demographic, and biological characteristics data. The resulting well-annotated biorepository is rapidly growing, adding opportunities for genetic and molecular profiling to better explore the linkage between biospecimens and clinical data throughout the patient treatment journey. In addition, various administrative register data and electronic medical records independent of the study hypothesis were accessed in the present investigation. This type of data almost completely avoids the possibility that selection, information, and recall bias influence the results, ensuring observation validity and minimizing participant loss at follow-up. Furthermore, the present prospective cohort study was performed in collaboration with a Clinical Research Center and Department of Obstetrics and Gynecology within the Shengjing Hospital of China Medical University. Thus, a large patient population from a single institution minimized treatment and clinical data collection heterogeneity. In addition, the quality of study design, measurement, and evaluation was guaranteed with the help of staff from the clinical research center. Of note, the study included OC patients who were treated using different measures, which means that the present study was able to provide an opportunity to clarify the progression of OC and explore different therapeutic methods.

The potential weaknesses of the OOPS include the fact that the self-reported data for some variables, such as lifestyle factors and occupational exposure, may introduce recall bias. Apart from that, some information on clinical outcome prognoses relies on inpatient medical, readmission, or outpatient records. As a result, some complications and other related events may be underreported and underestimated. In addition, the sample size is small in current analysis. However, the OOPS study is an on-going cohort, more and more OC patients will be recruited in our cohort. Finally, a single-institution patient population and its results may not be generalizable to all patients. Nevertheless, collaborations with a wider range of institutions are planned in order to create a cooperation network.

## Conclusion

In conclusion, the OOPS study collected detailed and comprehensive clinical, demographic, and biological characteristics data, which added opportunities to better explore the linkage between biospecimens and clinical data throughout the patient treatment journey. More research will be conducted on the prognosis of ovarian cancer.

## Data Availability Statement

The original contributions presented in the study are included in the article/supplementary material, further inquiries can be directed to the corresponding author/s.

## Ethics Statement

The studies involving human participants were reviewed and approved by Shengjing hospital of China Medical University. The patients/participants provided their written informed consent to participate in this study.

## Author Contributions

T-TG, Y-HZ, and Q-JW contributed to the study design. T-TG, SY, X-HH, XQ, and SG collection of data. F-HL and Y-FW analysis of data. T-TG, F-HL, Y-SL, H-LX, Y-FW, and Q-JW wrote the first draft of the manuscript and edited the manuscript. All authors read and approved the final manuscript.

## Funding

This work was supported by the National Key R&D Program of China (No. 2017YFC0907404 to Y-HZ), the Natural Science Foundation of China (No. 82073647 to Q-JW, No. 81602918 to Q-JW, and No.82103914 to T-TG), LiaoNing Revitalization Talents Program (No. XLYC1907102 to Q-JW), Shenyang high level innovative talents support program (No. RC190484 to Q-JW), 345 Talent Project of Shengjing Hospital of China Medical University (Q-JW and T-TG), and Clinical Research Cultivation Project of Shengjing hospital to SG.

## Conflict of Interest

The authors declare that the research was conducted in the absence of any commercial or financial relationships that could be construed as a potential conflict of interest.

## Publisher's Note

All claims expressed in this article are solely those of the authors and do not necessarily represent those of their affiliated organizations, or those of the publisher, the editors and the reviewers. Any product that may be evaluated in this article, or claim that may be made by its manufacturer, is not guaranteed or endorsed by the publisher.
